# lncRNA XIST Interacts with Regulatory T Cells within the Tumor Microenvironment in Chronic Hepatitis B-Associated Hepatocellular Carcinoma

**DOI:** 10.5146/tjpath.2023.13161

**Published:** 2024-05-18

**Authors:** Burcin Pehlivanoglu, Anil Aysal, Cihan Agalar, Tufan Egeli, Mucahit Ozbilgin, Tarkan Unek, Ilkay Tugba Unek, Ilhan Oztop, Safiye Aktas, Ozgul Sagol

**Affiliations:** Department of Molecular Pathology, Graduate School of Health Sciences, Dokuz Eylul University, Izmir, Turkey; Department of Pathology, Dokuz Eylul University, Faculty of Medicine, Izmir, Turkey; Department of General Surgery, Dokuz Eylul University, Faculty of Medicine, Izmir, Turkey; Department of Medical Oncology, Dokuz Eylul University, Faculty of Medicine, Izmir, Turkey; Department of Molecular Pathology, Graduate School of Health Sciences, Dokuz Eylul University, Izmir, Turkey; Department of Basic Oncology, Dokuz Eylul University, Institute of Oncology, Izmir, Turkey

**Keywords:** Hepatocellular carcinoma, lncRNA, Inflammation, Chronic hepatitis B, XIST, CD25, Regulatory

## Abstract

***Objective: *** Alterations in the expression of several long non-coding RNAs (lncRNAs) have been shown in chronic hepatitis B-associated hepatocellular carcinoma (CHB-HCC). Here, we aimed to investigate the association between the expression of inflammation-associated lncRNA X-inactive specific transcript (*XIST*) and the type of inflammatory cells within the tumor microenvironment.

***Material and Methods:*** Twenty-one consecutive cirrhotic patients with CHB-HCC were included. *XIST* expression levels were investigated on formalin-fixed paraffin-embedded (FFPE) tumoral and peritumoral tissue samples by real-time polymerase chain reaction (RT-PCR). Immunohistochemical staining for CD3, CD4, CD8, CD25, CD163, CTLA4, and PD-1 were performed. The findings were statistically analyzed.

***Results: *** Of the 21 cases, 11 (52.4%) had tumoral and 10 (47.6%) had peritumoral *XIST* expression. No significant association was found between the degree of inflammation and *XIST* expression. The number of intratumoral CD3, CD4, CD8 and CD20 positive cells was higher in *XIST*-expressing tumors, albeit without statistical significance. Tumoral and peritumoral *XIST* expression tended to be more common in patients with tumoral and peritumoral CD4high inflammation. The number of intratumoral CD25 positive cells was significantly higher in *XIST*-expressing tumors (p=0.01). Tumoral *XIST* expression was significantly more common in intratumoral CD25high cases (p=0.04). Peritumoral *XIST* expression was also more common among patients with CD25high peritumoral inflammation, albeit without statistical significance (p=0.19).

***Conclusion:*** lncRNA *XIST* is expressed in CHB-HCC and its expression is significantly associated with the inflammatory tumor microenvironment, particularly with the presence and number of CD25 (+) regulatory T cells. In vitro studies are needed to explore the detailed mechanism.

## INTRODUCTION

Chronic hepatitis B (CHB) is a well-known risk factor for development of hepatocellular carcinoma (HCC) (1). In CHB, the integration of the hepatitis B virus (HBV) results in genomic instability and mutations in hepatocytes, and persistent inflammation causes activation of inflammation-associated pathways (2–7). Molecular alterations caused by hepatitis viruses may occur both in the coding and non-coding regions of the host genome (8). Long non-coding RNAs (lncRNAs), which are non-coding transcripts of more than 200 nucleotides, have been shown to be involved in CHB-associated HCC (CHB-HCC) pathogenesis (9–13).

We recently investigated inflammation-associated lncRNA expression in formalin-fixed paraffin-embedded (FFPE) tissue samples (chronic viral hepatitis (CVH)-associated HCC, peritumoral cirrhotic parenchyma, nontumoral cirrhotic CVH parenchyma, and normal liver) using a real-time polymerase chain reaction (RT-PCR) array panel of 84 inflammation-associated lncRNAs, and then, based on the RT-PCR array results, we performed RT-PCR assay testing to compare expression patterns of 7 inflammation-associated lncRNAs between CVH-HCC, peritumoral cirrhotic parenchyma, and nontumoral CVH samples as a second step (manuscript in preparation). One of the studied lncRNAs was lncRNA X-inactive specific transcript (*XIST*), which we found to be associated with the presence of negative prognosticators in CVH-HCC (manuscript in preparation). *XIST *is expressed in all female somatic cells, especially during the early phases of development, and in germ cells in males but its expression in neoplastic and nonneoplastic diseases is variable and is considered to result from abnormal activation of X chromosome-related genes during pathological processes (14–16).

In addition to its significant association with negative prognosticators, we also observed that peritumoral *XIST* expression was related with neutrophil predominant inflammation in peritumoral cirrhotic parenchyma. Thus, we aimed to investigate the association between the expression of inflammation-associated lncRNA *XIST* and the type of inflammatory cells within the tumor microenvironment, i.e., within the HCC focus and nearby cirrhotic parenchyma, in the current study.

## MATERIAL and METHODS

### Case Selection and Collection of Clinicopathological Data

The study was approved by the institutional ethics committee (2019/05-70). A total of 21 consecutive cirrhotic patients with CHB-HCC who had underwent liver transplantation without prior interventional treatment were included. Clinicopathological data were retrieved from the hospital records. Hematoxylin-eosin-stained slides of all cases were re-evaluated, and the degree and type of inflammation in neoplastic and peritumoral non-neoplastic tissue were noted.

### Immunohistochemistry

Areas representing the highest degree of inflammation were marked on hematoxylin-eosin-stained slides of tumoral and peritumoral tissue samples. Tissue micro-array (TMA) blocks composed of tumoral and nontumoral tissue cores of 5 mm were created. Then, sections of 3 microns were taken from the TMA blocks. Immunostaining for CD3 (*CONFIRM anti-CD3 (2GV6) Rabbit Monoclonal Primary Antibody, Ventana, AZ, USA*), CD4 (*CONFIRM anti-CD4 (SP35) Rabbit Monoclonal Primary Antibody, Ventana, AZ, USA*), CD8 (*CONFIRM Anti-CD8 (SP57)* *Rabbit Monoclonal Primary Antibody, Ventana, AZ, USA*) CD20 (*CONFIRM anti-CD20 (L26)* *Primary Antibody, Ventana, AZ, USA)*, CD25 (*CD25 (4C9) Mouse Monoclonal Antibody, Cell Marque, CA, USA*), CD163 (*CD163 (MRQ-26) Mouse Monoclonal Antibody, Cell Marque, CA, USA)*, CTLA4 (*CTLA-4 Antibody (F-8): sc-376016, Monoclonal, Santa Cruz Biotechnology, USA) *and PD-1 (*PD-1 (NAT105) Mouse Monoclonal Antibody, Cell Marque, CA, USA)* were performed following deparaffinization, rehydration, and antigen retrieval procedures on a fully automated immunostaining system *(Ventana BenchMark XT, AZ, USA). *Positive control (tonsil tissue) was also run with each group of slides.

**Figure 1:**
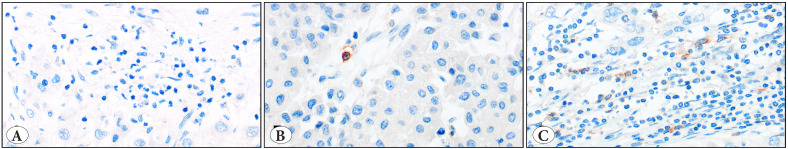
Immunohistochemical evaluation. The number of the positive cells was counted in one high power field and the median was determined. In addition to negative/positive categorization, values less than the median value were grouped as “low”, and values more than the median value were grouped as “high” for each marker except CTLA-4, which was evaluated either as negative or positive. **A)** Intratumoral CD25negative, **B)** Intratumoral CD25low, and **C)** Intratumoral CD25high.

One high power field containing the highest number of positive cells were photographed, and the number of the positive cells were counted. The median was determined for each group. In addition to negative/positive categorization, values less than the median value was grouped as “low”, and values more than the median value were grouped as “high” for each marker except for CTLA4 (Figure 1). CTLA4 staining was grouped as “negative” or “positive”.

### Measurement of *XIST* Expression

*XIST* primary assay (*RT² QPCR Primer Assay, Qiagen, Germany*) was used to detect *XIST* expression levels on FFPE tissue samples by RT-PCR. Hematoxylin-eosin-stained slides were reviewed to select representative tumor and peritumoral cirrhotic parenchyma (within 1 cm distance from the HCC focus) areas. Selected areas were marked. Then, five sections of 7 microns from the selected areas were taken and placed into Eppendorf tubes. RNA extraction was done according to manufacturer’s recommendations using a kit for FFPE tissues (*RNEASY FFPE Kit, Qiagen, Germany*). cDNA synthesis and preamplification were then done using a suitable kit (*RT² PREAMP cDNA Synthesıs Kit, Qiagen, Germany*) before preparation for PCR. *RT2 SYBR Green Mastermix* *(Qiagen, Germany)* was used during sample preparation. Glyceraldehyde 3-phosphate dehydrogenase (GAPDH) was used as the reference gene. The Ct value was determined for each case and the expression status was interpreted qualitatively, i.e., as expressed or not-expressed.

### Statistical Analysis

Statistical analyses were performed using SPSS ver. 24 (*IBM, USA*). In addition to frequency analyses, the chi-square test was used to compare categorical variables and Kruskal-Wallis’s test was used for comparisons between 2 or more groups. The association between the parameters was sought using the Pearson or Spearman correlation test. p<0.05 was considered statistically significant.

## RESULTS

### Clinicopathological Features

All cases had histopathologically verified cirrhosis. Two cases were female (F:M = 0.10). The median age was 58 ± 4.66 years (range 48-64 years). Six patients had hepatitis D virus (HDV) co-infection. The mean tumor diameter was 3.44 ±1.33 cm (range 0.9-6 cm), and 10 patients (47.6%) had multifocal tumor. No patient had portal vein thrombosis. Other clinicopathological characteristics are summarized in Table 1.

**Table 1:** Clinicopathological characteristics.

Tumor grade Grade 1 Grade 2 Grade 3 Grade 4	4 (19%) 14 (66.7%) 1 (4.8%) 2 (9.5%)
pT stage pT1 pT2 pT3 pT4	11 (52.4%) 7 (33.3%) 1 (4.8%) 2 (9.5%)
PN stage pN0 pN1	20 (95.2%) 1 (4.8%)
Venous invasion Present Absent	5 (23.8%) 16 (76.2%)
The degree of intratumoral inflammation Score 1 (Scattered inflammatory cells) Score 2 (Mild inflammation) Score 3 (Moderate inflammation) Score 4 (Substantial inflammation)	4 (19%) 10 (47.6%) 2 (9.5%) 5 (23.8%)
The type of intratumoral inflammation Mixed with abundant lymphocytes, neutrophils, plasma cells Neutrophil predominant mixed Lymphoplasmacytic predominant with occasional neutrophils Lymphoplasmacytic predominant with rare neutrophils	5 (23.8%) 12 (57.1%) 4 (19%) 0
The degree of peritumoral inflammation Score 1 (Mild inflammation) Score 2 (Moderate inflammation) Score 3 (Severe inflammation)	7 (33.3%) 8 (38.1%) 6 (28.6%)
The type of intratumoral inflammation Mixed with abundant lymphocytes, neutrophils, plasma cells Neutrophil predominant mixed Lymphoplasmacytic predominant with occasional neutrophils Lymphoplasmacytic predominant with rare neutrophils	8 (38.1%) 4 (19%) 7 (33.3%) 2 (9.5%)

### Immunophenotype of the Tumor Microenvironment

The number of the CD3, CD4, CD8 and CD20 positive cells were significantly lower in the tumor compared to the peritumoral cirrhotic parenchyma (p<0.05; Table 2). There was moderate correlation between intratumoral and peritumoral CD25 positive cell counts (r=0.57; p=0.008, Pearson correlation test). The intratumoral CD25 positive cell count was also significantly correlated with intratumoral CD3, CD4, CD8, CD20, and CTLA4 positive cell counts (p<0.05).

**Table 2:** Comparison of immunohistochemical cell counts between the tumor and peritumoral parenchyma samples.

	**Intratumoral (median ± SD)**	**Peritumoral (median ± SD)**	**p**
**CD3**	69 ± 48.93 (Range: 6-169)	139.5 ± 38.43 (Range: 51-210)	**<0.001**
**CD4**	46 ± 37.53 (Range: 3-126)	76.5 ± 27.56 (Range: 31-121)	**0.013**
**CD8**	20 ± 24.87 (Range: 0-81)	55 ± 25.90 (Range: 6-105)	**0.005**
**CD20**	22.5 ± 31.76 (Range: 0-114)	60 ± 59.43 (Range: 3-240)	**0.006**
**CD163**	34 ± 19.93 (Range: 3-94)	36 ± 10.89 (Range: 11-55)	0.47
**CD25**	1 ± 4.14 (Range: 0-13)	3 ± 2.09 (Range: 1-8)	0.56
**CTLA-4**	0 ± 1.72 (Range: 0-7)	0 ± 1.60 (Range: 0-5)	0.85
**PD-1**	1 ± 3.35 (Range: 0-14)	1 ± 7.63 (Range: 0-33)	0.45

The tumoral CD8 score was significantly associated with the peritumoral CD3 score (p=0.028). However, no other significant difference or association was found between the tumoral and peritumoral groups regarding the distribution of the inflammatory cell subgroups (p>0.05).

### *XIST* Expression Analysis

Out of the 21 cases, 11 (52.4%) had tumoral and 10 (47.6%) had peritumoral *XIST* expression, and there was strong correlation between tumoral and peritumoral *XIST* Ct values (r=0.90; p=0.037, Pearson test), albeit without significant correlation between tumoral and peritumoral expression levels (p=0.76). There was peritumoral *XIST* expression in 4 patients with neutrophil predominant peritumoral inflammation (4/4 vs. 6/17; p=0.02). No significant association was found between the overall degree of inflammation and *XIST* expression.

### Association Between *XIST* Expression and the Immunophenotype of the Inflammatory Cells

The number of intratumoral CD3, CD4, CD8, and CD20 positive cells was higher in *XIST*-expressing tumors but the difference did not reach statistical significance (Table 3). Peritumoral *XIST* expression was significantly more frequent among patients with tumoral CD3high (8/12 vs. 2/7; p=0.044) and CD4high (8/11 vs. 2/10; p=0.016) patients. Tumoral *XIST* expression was more common in patients with tumoral CD4high inflammation and peritumoral *XIST* expression was more common in patients with peritumoral CD4high inflammation, albeit without statistical significance (7/11 vs. 4/10; p=0.27 and 5/8 vs. 4/12; p=0.19, respectively).

**Table 3:** Association between the phenotype of the inflammatory cells and XIST expression.

		**Intratumoral (median ± SD)**		**p**	**Peritumoral (median ± SD)**		**p**
		***XIST*** **expression**	**No** ***XIST*** **expression**		***XIST*** **expression**	**No** ***XIST*** **expression**	
**Intratumoral**	**CD3**	90 ± 58.06	58 ± 32.83	0.17	88 ± 47.11	50 ± 46.34	0.1
	**CD4**	50 ± 44.06	34.5 ± 27.58	0.25	62.5 ± 33.09	33 ± 36.22	0.052
	**CD8**	30 ± 27.31	18 ± 21.82	0.32	25.5 ± 19.63	13 ± 29.61	0.65
	**CD20**	31 ± 41.04	18.5 ± 15.8	0.19	22.5 ± 33.08	22.5 ± 31.76	0.63
	**CD163**	32 ± 24.43	37 ± 14.51	0.65	34 ± 26.60	34 ± 14.22	0.8
	**CD25**	**3 ± 4.87**	**0.5 ± 0.69**	**0.011**	2 ± 5.27	1 ± 1.79	0.056
	**CTLA-4**	0 ± 2.07	0 ± 1.33	0.78	0.5 ± 2.31	0 ± 0.64	0.13
	**PD-1**	2 ± 1.78	1 ± 4.59	0.59	1 ± 4.27	1 ± 2.45	0.88
**Peritumoral**	**CD3**	135 ± 23.75	141 ± 50.54	0.99	139 ± 42.82	140 ± 32.98	0.20
	**CD4**	72.5 ± 29.82	83 ± 26.73	0.99	89 ± 33.13	75 ± 23.45	0.66
	**CD8**	38 ± 27.27	61.50 ± 19.73	0.052	50.5 ± 27.17	62 ± 25.43	0.51
	**CD20**	64 ± 45.73	60 ± 72.29	0.39	60 ± 42.71	64 ± 72	0.42
	**CD163**	38 ± 11.87	34 ± 10.31	0.87	31 ± 10.91	38 ± 10.46	0.19
	**CD25**	2.5 ± 2.57	3 ± 1.54	0.53	4 ± 2.55	2 ± 1.4	0.1
	**CTLA-4**	0 ± 1.55	0 ± 1.72	0.60	0 ± 2.10	0 ± 1.07	0.8
	**PD-1**	1 ± 9.67	1 ± 4.92	0.67	1 ± 10.06	1 ± 4.83	0.56

The number of intratumoral CD25 positive cells was significantly higher in *XIST-*expressing tumors (p=0.01) (Table 3) (Figure 1). Tumoral *XIST* expression was significantly more common in intratumoral CD25high cases compared to CD25low and CD25negative cases (7/8 vs. 2/6 vs. 2/7; p=0.04). There was moderate negative correlation between tumoral *XIST* Ct values and intratumoral CD25 counts, and strong negative correlation between tumoral *XIST* Ct values and peritumoral CD25 counts (r=-69; p=0.018 and r=-0.84; p=0.002, respectively, Pearson test). The intratumoral *XIST* expression level was moderately correlated with the intratumoral CD25score (r=0.52; p=0.014, Spearman correlation test).

Peritumoral *XIST* expression was also more common among patients with CD25high peritumoral inflammation, albeit without statistical significance (5/8 vs.4/12; p=0.19), and no significant correlation was found between peritumoral *XIST* expression level and peritumoral CD25 count (p=0.09).

There was a moderate negative correlation between peritumoral *XIST* Ct values and peritumoral CTLA-4 positive cell counts (r=-67; p=0.031, Pearson test), although there was no significant correlation between the peritumoral *XIST* expression level and the peritumoral CTLA4 score (p>0.05).

## DISCUSSION

In this study, we evaluated the association between the expression of lncRNA *XIST* and the type of inflammatory cells within the tumor and peritumoral cirrhotic liver parenchyma.

Our study is one of the few studies on *XIST* expression in hepatocellular carcinoma that was conducted using tissue samples (17–22). Overall, about half of the cases had tumoral and/or peritumoral *XIST* expression. Tumoral and peritumoral *XIST* Ct values were strongly correlated. Although our study group is not appropriate for gender comparison regarding *XIST *expression considering there were only 2 women, 9/11 cases showing tumoral expression were male, and considering *XIST *is not normally expressed in somatic cells in males (14–16), its expression in CHB-HCC and peritumoral liver tissues is considered pathological.

*XIST* is a critical regulator in X chromosome inactivation (hence the name), but it has also been shown to be involved in many biological and pathological processes, including cancer, inflammation, and cardiac and neurological diseases (23,24). *XIST* has been demonstrated to alter the inflammatory response via the NF-κB pathway, which is also an important pathway in development of inflammation-associated HCC (5,25,26). All 4 patients with neutrophil predominant peritumoral inflammation expressed *XIST* in the peritumoral parenchyma in the current study. There is limited data on the association between *XIST* expression and neutrophilic inflammation in the literature. While Shenoda et al. have claimed that *XIST* weakens the acute inflammatory response in female cells, *XIST* silencing has been demonstrated to reduce neutrophil extracellular trap formation in another recent study (25,27). These different findings may simply reflect the fact that the effect of lncRNAs is generally dependent of other factors such as microRNA expression. In any case, we believe that the association between *XIST* expression and neutrophilic infiltration deserves further investigation.

The number of the CD3 positive cells was significantly lower in the tumor compared to peritumoral cirrhotic parenchyma. However, curiously, we found a significant association between peritumoral *XIST* expression and tumoral CD3 positive cell count. In addition, the number of the intratumoral CD3, CD4, CD8 and CD20 positive cells were higher in *XIST* expressing tumors, although no statistical significance could be reached. These findings indicate that *XIST* expression induces inflammation, particularly T cell proliferation, in the tumor microenvironment, most likely due to stimulation of cytokine production as previously demonstrated (28,29).

We also observed that tumoral and peritumoral *XIST* expression were more common in patients with CD4high inflammation, albeit without statistical significance, probably due to the small number of the patients in the study group. However, this finding seems to be consistent with the literature. Recently, high-expression of *XIST *in CD4-positive cells and natural killer cells has been suggested to trigger the proliferation of naïve CD4 (+) T cells in primary biliary cholangitis (30). In another study, high *XIST* expression has been found to be associated with the CD4-positive T cell level in systemic lupus erythematosus patients (31).

CD4+CD25+ regulatory T cells are suppressor T cells and the presence of IL-2 is required for their survival (32–34). Similar to our study, CD4+CD25+ regulatory T cells have been shown to increase in CHB and CHB-HCC, and they are thought to inhibit the antitumoral immune response, resulting in a tumor promoting environment (35–39). In addition, one of the major findings in the current study was the significant association between the tumoral *XIST* expression and the number of the intratumoral CD25-positive inflammatory cells. *XIST* was more frequently expressed in intratumoral CD25high cases. Peritumoral *XIST* expression also tended to be more frequent in patients with CD25high peritumoral inflammation, albeit without statistical significance. These findings suggest that increase in CD25-positive T cells may be the result of *XIST* expression. Activated mature T cells have been shown to restore *XIST* expression, and the *XIST* location pattern of regulatory T cells has been demonstrated to differ dependent of the stimulation time in mice (40). There is also evidence of *XIST *regulating the proliferation of T helper subsets (41,42). Therefore, there may be an interaction between *XIST*-expressing hepatocytes and T cells within the CHB-HCC microenvironment, causing the activation of CD25-positive regulatory T cells. Moreover, NF-κB has been claimed to control IL-2 expression during T cell development (43). Considering that *XIST* acts via the NF-κB pathway (see above), it may be facilitating IL-2 expression, and thus CD25 expression in T cells. However, in vitro studies are required to demonstrate the possible direct association between *XIST* expression and CD25 positive T cells.

The most important limitation of our study was the small number of patients. However, we believe that this study provides essential findings for possible future in vitro studies on the association between *XIST *expression and regulatory T cells.

In conclusion, lncRNA *XIST* is expressed in CHB-HCC and induces inflammation, and its expression is significantly associated with the inflammatory tumor microenvironment, particularly with CD25 (+) regulatory T cells. In vitro studies are needed to establish the detailed mechanism and contributing pathways of this association.

## Funding

This study was supported by the Research Fund of the Dokuz Eylul University (Project Number: 2021.KB.SAG.019) and Turkish Association for the Study of the Liver (TASL).

## Conflict of Interest

The authors have no conflicts of interest to declare.
